# Optimizing the mucosal frontier: longevity and epithelial barriers in prime-boost mRNA vaccine strategies

**DOI:** 10.1186/s12967-026-08532-0

**Published:** 2026-06-26

**Authors:** Xinru Tang, Fang Jiang, Shiwei Ni

**Affiliations:** 1 The First Affiliated Hospital of Dalian Medical University, Dalian, China; 2 Central Hospital of Dalian University of Technology, Dalian, China; 3 Dalian University of Technology, Dalian, China

**To the Editor:**

We read with great interest the study by Zhang and colleagues [1], which evaluates an intramuscular (IM) prime and intranasal (IN) boost immunization strategy for an mRNA-LNP vaccine. The authors convincingly demonstrate that this sequential regimen significantly enhances both systemic IgG responses and localized mucosal IgA secretion against SARS-CoV-2 in a murine model [1]. By analyzing the B-cell receptor (BCR) repertoire and clonal convergence, the study provides a robust translational foundation for generating respiratory mucosal immunity [1]. While these findings offer a promising blueprint for next-generation vaccines, we believe several biological barriers and physiological variables must be addressed to advance this strategy toward successful human clinical trials.

The first critical consideration involves the long-term maintenance and structural homing of resident memory B cells (BRM) within the respiratory mucosa. Zhang et al. observe a high hypermutation frequency and robust germinal center responses at day 56 post-immunization [1]. However, the respiratory mucosa is an environment under constant environmental stress and cellular turnover [2]. In clinical translation, animal-derived short-term BRM expansion does not automatically guarantee multi-year protection in humans. Without specific signals, such as the localized upregulation of CXCR3 or alpha-4-beta-7 integrins, intranasally boosted memory pools may gradually decline or migrate away from the upper respiratory tract [3]. Characterizing the longitudinal survival kinetics of these tissue-resident pools is essential to predict the durability of protection against viral variants.

Furthermore, the translational execution of intranasal boosting must reconcile the immense physical barrier presented by the human mucus layer and ciliary clearance mechanisms. In mice, volume-to-surface area ratios allow topically applied mRNA-LNPs to easily reach the epithelial border [1]. In contrast, the human nasal passage possesses a thick, dynamic mucus blanket that traps foreign particles [4]. Normal ciliary beating rapidly clears standard lipid nanoparticles into the gastrointestinal tract within minutes [4]. This rapid clearance drastically limits the time available for epithelial uptake and subsequent mRNA translation. Consequently, defining the optimal kinetic formulation to penetrate this fluid barrier without causing systemic leakage remains a significant challenge.

To optimize the clinical translation of this prime-boost paradigm, we suggest two programmatic engineering strategies: Development of Mucopenetrating Matrix Formulations: Future iterations should chemically modify the outer LNP shell, such as incorporating low-molecular-weight polyethylene glycol (PEG) coatings, to minimize steric hindrance by mucin fibers and prolong epithelial contact time. Rigorous Safety Profiling of Respiratory Adjuvants: Because the nasal mucosa is directly connected to the olfactory bulb, intranasal administration of lipid matrices requires stringent neuro-immunological safety assays [4]. Preclinical safety models must prove that repeated IN boosting does not trigger chronic inflammatory responses or epithelial disruption in the upper respiratory tract.

In conclusion, Zhang and colleagues [1] provide an elegant and logically sound strategy for overcoming the limitations of purely systemic vaccination. By addressing the physical constraints of the mucosal barrier and the long-term survival of resident immune cells, the translational vaccine community can successfully advance this promising platform from murine models to human clinical realities.

## Data Availability

No specific data or materials were generated or analyzed in this letter.
